# Decrease in Lipid Droplets in Adrenal Cortex of Male Wistar Rats after Chronic Exposure to Energy Drinks

**DOI:** 10.3390/medicina54050090

**Published:** 2018-11-19

**Authors:** Michał K. Zarobkiewicz, Mateusz M. Woźniakowski, Ewelina Wawryk-Gawda, Mirosław A. Sławiński, Paweł Halczuk, Agnieszka Korolczuk, Barbara Jodłowska-Jędrych

**Affiliations:** 1 Chair and Department of Histology and Embryology with Experimental Cytology Unit, Medical University of Lublin, 20-080 Lublin, Poland; m.wozniakowski16@gmail.com (M.M.W.); ewelinawawrykgawda@umlub.pl (E.W.-G.); miroslaw.slawinski92@gmail.com (M.A.S.); halczuk.pawel@gmail.com (P.H.); barbara.jodlowska-jedrych@umlub.pl (B.J.-J.); 2 Chair and Department of Clinical Patomorphology, Medical University of Lublin, 20-090 Lublin, Poland; agnieszka.korolczuk@umlub.pl

**Keywords:** energy drinks, Wistar, rats, adrenal glands, caffeine, taurine

## Abstract

*Background and objectives*: Energy drinks are popular non-alcoholic beverages. They are consumed in large amounts, mainly by active, young people. Although they are easily accessible and marketed as safe, numerous cases of adverse effects have been published, including cardiac arrest, arrythmias, acute hepatitis, and renal failure. The aim of the current study is the assessment of energy drink influence on the histological structure of adrenal cortex in rats. *Material and Methods*: 15 male young Wistar rats were equally divided into three groups: control (C), experimental (E) and reversibility control (RC). C group received water and standard rodent food *ad libitum* while both E and RC groups had additionally unlimited access to energy drinks. C and E groups were decapitated after 8 weeks and RC was given another 8 weeks without energy drinks. Adrenal glands were embedded in paraffin blocks and 5 μm slides were prepared and stained according to standard H&E and Masson’s trichrome protocols. Additionally, immunohistochemical stainings against Ki-67, p53, CTGF and caspase-3 were prepared. *Results*: Decreased vacuolization and numerous pyknotic nuclei were noted in E and RC groups. Overexpression of caspase-3 was noted both subcapsular in zona glomerulosa and along sinusoids in zona fasciculata. Increased collagen deposition in zona glomerulosa and zona fasciculata of E and RC was observed. Insular and irregular overexpression of CTGF was noted. The overall picture of CTGF expression matched the Masson’s trichrome. No significant difference was observed in Ki-67 expression. *Conclusions*: The results of the current study suggest that the stimulation is so intense that it causes significant damage to adrenal cortical cells, resulting in their apoptosis. It seems, however, that the observed effects are at least partially reversible.

## 1. Introduction

Energy drinks were introduced in Japan more than five decades ago [1]. Since then a large worldwide market has emerged. In 2012 it was estimated to be worth 12.5 billion USD. As the energy drinks are advertised as boosters or stimulants, large amounts of them are consumed, mainly by young people [2]. Numerous scientific publications concerning the influence of energy drinks on human health have been published over the last few years. Unfortunately, the collected data are still incomplete. Numerous cases of adverse effects have been published, among them: severe arrhythmias [3], tachycardia [4,5], hypertension [4], acute renal failure [6], tonic-clonic seizures [7,8,9], acute hepatitis [10], cardiac arrest [5,11] and myocardial infarction [12,13]. Jonjev Z. S. and Bala G. suggest that energy drinks can provoke aortic dissection [14]. Polat N. et al. reported a case of a 13-year old boy with spontaneous coronary artery dissection, probably caused by energy drinks ingestion [13]. Avci S. et al. reported a case of energy drink related death [15].

There is no strict definition of what an energy drink is, but it is commonly associated with a non-alcoholic beverage with high caffeine content (usually 32 mg/100 mL), a large dose of sugar (or sweeteners) and additional ingredients (taurine, guarana, glucuronolacton, vitamins). The current study used a typical energy drink with 32 mg/100 mL caffeine and 0.4% taurine content.

Caffeine, the main ingredient of energy drinks, is known to be capable of glucocorticoid stimulation [16]. A hypothesis has therefore been drawn that chronic energy drinks consumption may lead to an adrenal cortex overstimulation with visible structural changes. The aim of our study is to analyze the structure of rat adrenal cortex after chronic consumption of energy drinks.

## 2. Materials and Methods

Research was conducted on 15 young male Wistar rats (mean body weight of 155.4 g), divided equally into three subgroups: control (C), experimental (E) and reversibility control (RC). All groups had equal access to food and water, the control group also had unlimited access to water, the experimental one—to water and energy drinks, and the reversibility control one—to water and energy drinks for 8 weeks and to water for further 8 weeks. All the rats were sacrificed by decapitation—the control group and the experimental group were decapitated after 8 weeks, that is after the last dose of energy drinks. The reversibility control group was observed for another 8 weeks without supplementation of energy drinks, and after a total of 16 weeks was also decapitated. Adrenal glands were collected, fixed in 10% formalin, and afterwards embedded in paraffin blocks.

### 2.1. Energy Drink Composition

Energy drink, used in the current study, contained 32 mg caffeine per 100 mL, taurine (0.4%), and inositol (0.02%). It was also enriched with vitamins: niacin (7.92 mg/100 mL), pantothenic acid (1.98 mg/100 mL) and B6 (2 mg/100 mL).

### 2.2. Histological Staining and Analysis

5 μm thick slices were prepared and stained according to standard H&E and Masson’s trichrome protocol. The evaluation was made first under a light microscope and further also using a microscope with a digital camera (Olympus BX4, London, UK). Fibrosis was assessed by both standard visual evaluation and computed analysis. The latter was performed by color deconvolution and consequent optical density (OD) calculation in Fiji (University of Wisconsin-Madison, WI, USA), as proposed by Shi et al. [17].

A series of immunohistochemical stainings was performed. Monoclonal antibodies directed against Ki-67 (AbD Serotec, Kidlington, UK, AbD02531, dilution: 1:100), caspaze 3 (Sigma-Aldrich, Saint Louis, MO, USA, WH0000836M2, dilution: 1:40), p53 (Sigma-Aldrich, Saint Louis, MO, USA, P5813, dilution: 1:200) and CTGF (AbD Serotec, Kidlington, UK, AHP1278, dilution: 1:500, connective tissue growth factor) were used. All antibodies were previously successfully used for rat or mouse tissue staining [18,19]. The data on the CTGF has not yet been published. A protocol similar to proposed by Jędrych et al. [20] was used. Two alternations to the method were introduced—the incubation with the primary antibody was performed for 60 minutes at room temperature instead of overnight incubation at 4 °C, and in the case of CTGF, antigenic sites were exposed by incubation with Proteinase K (Sigma-Aldrich, Saint Louis, MO, USA) for 10 minutes at room temperature.

CellSens (Olympus, Waltham, MA, USA) software was used for detailed measurements of cortex layer thickness as well as the area occupied by lipid droplets. Study design was approved by the Ethics Committee of the Medical University of Lublin (Approval No. 6a/2013).

### 2.3. Statistical Analysis

The collected statistical data were analyzed with Statistica 10 (StatSoft, Tulsa, OK, USA). The Shapiro Wilk test was used for determination if the distribution was normal or not. Brown Forsyth test was employed to test if the variances were homogeneous. The statistical significance was measured with *F* variance analysis test (vacuoles as percentage of total area) and Kruskal-Wallis test for the remaining analyses. The level of significance was set at *p* < 0.05.

## 3. Results

Rats in the experimental and reversibility control groups drank about 0.190 mL of energy drinks/g of body weight/day. Therefore, the mean daily caffeine consumption was approximately 9.45 mg per rat.

### 3.1. H&E Staining

**Figure 1:**
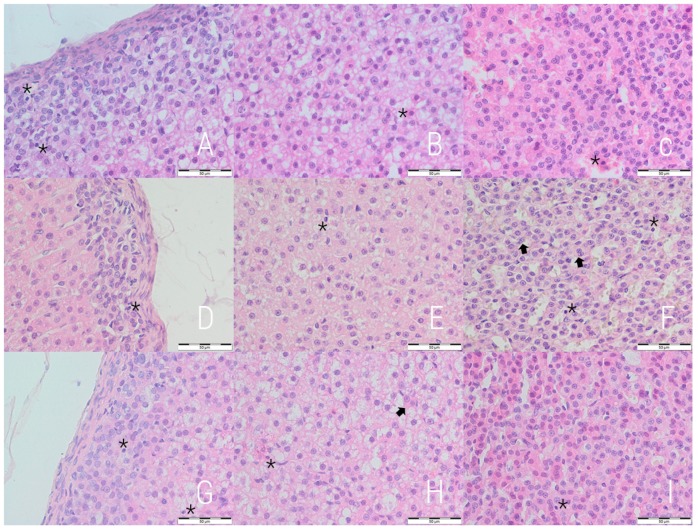
Adrenal glands 400× magnified; the control (**A**–**C**), the experimental (**D**–**F**) and reversibility control (**G**–**I**) groups. The first column shows the zona glomerulosa, second—zona fasciculata and third—zona reticularis. Differences in size and number of lipid droplets can be noted. Signs of decreased vacuolization are visible in zona fasciculata of the experimental (**E**) and reversibility control groups (**H**). Note pyknotic nuclei (*) and binucleated cells (**arrow**). H&E staining (bar = 50 µm).

The qualitative assessment revealed a general decrease in the content of lipid droplets in the experimental and reversibility control groups, especially pronounced in the former one. The lipid droplets were smaller and more numerous, cytoplasmic diffuse vacuolization of different intensity was also noted (Figure 1). Those vacuoles consisted of both micro- and macrovesicles. For quantitative evaluation, the contribution of lipid droplets to the total selected area was measured independently for each cortex layer. Decreased content of lipid droplets was noted in the E and RC groups in comparison to the C group in zona fasciculata, while an increase was noted in zona reticularis (Table 1).

A well-marked irregularity of cellular arrangement as well as nuclear heterochromy and irregularity were observed in the E and RC groups. Both groups had more numerous pyknotic nuclei (Figure 1). The highest values for all three cortex layers was observed for the experimental group, but the difference is statistically not significant (Table 2).

### 3.2. Masson’s Trichrome Staining

The initial evaluation revealed changes in the relative thickness of cortex layers, therefore, detailed measurements was performed (Table 3). An increase in the contribution of zona fasciculata was observed in both the experimental and reversibility control groups with the highest value in the former one. A decrease was noted in the case of zona glomerulosa in both the E and RC groups with a higher value in the E group. No significant differences in contribution of zona reticularis to total cortex thickness was noted.

**Figure 2:**
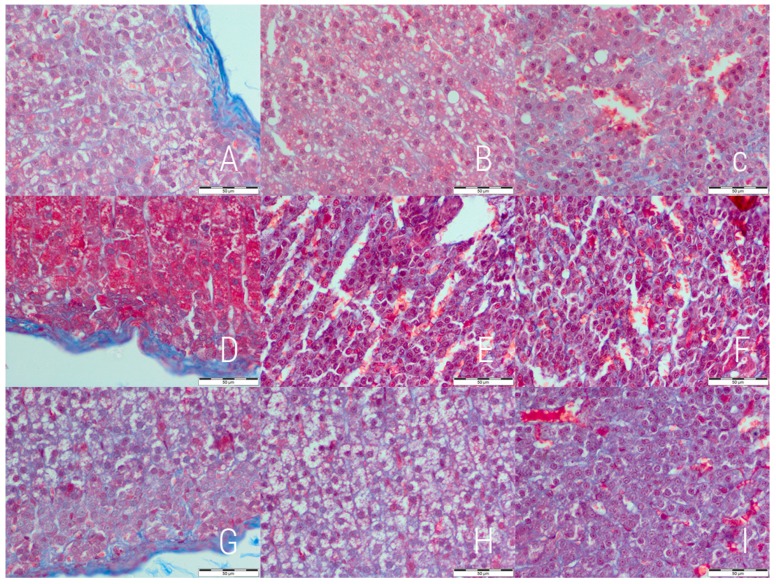
Adrenal glands 400× magnified; the control (**A**–**C**), experimental (**D**–**F**) and reversibility control (**G**–**I**) groups. The first column shows the *zona glomerulosa*, second—*zona fasciculata* and third—*zona reticularis*. Increased collagen deposition can be noted in *zona glomerulosa* and *zona fasciculata* of both experimental (**D**,**E**) and reversibility control (**G**,**H**) groups. Masson’s trichrome staining (bar = 50 µm).

The analysis of Masson’s trichrome stained slides revealed signs of increased collagen deposition in both the experimental and reversibility control groups in comparison to the control (Figure 2). The fibrosis features were mostly notable in zona glomerulosa and zona fasciculata. Observations were confirmed by computed analysis with optical density calculation (Table 4). The highest optical density was noted in zona glomerulosa and zona fasciculata of the experimental group. Adrenal capsule was evaluated separately—visual analysis revealed no significant differences in collagen deposition in this area. No capsular fibrosis was observed in the experimental groups, but the highest mean thickness was noted in the control group (18.86 ± 3.68 μm vs. 11.39 ± 2.82 μm in E and 12.74 ± 1.73 μm in RC group, *p* = 0.0025).

### 3.3. Immunohistochemical Staining

**Figure 3:**
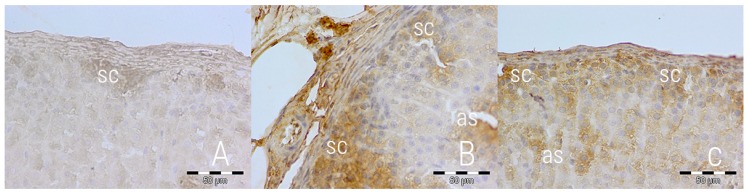
Caspase 3 expression in adrenal glands 400× magnified in the control (**A**), experimental (**B**) and reversibility control (**C**) groups. Visible focused subcapsular (**sc**) expression in *zona glomerulosa* and along sinusoids (**as**) in *zona fasciculata*. IHC staining (bar = 50 µm).

**Figure 4:**
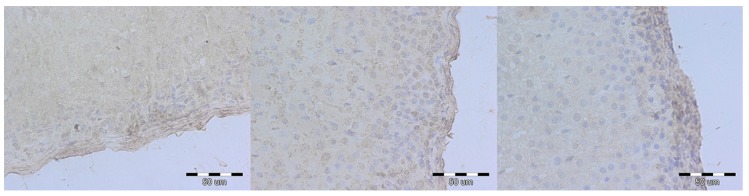
P53 expression in adrenal glands 400× magnified in the control (**A**), experimental (**B**) and reversibility control (**C**) groups. The expression of p53 is similarly low in all the groups within all three cortex layers. IHC staining (bar = 50 µm).

An immunohistochemical staining directed against caspase 3 was performed to further the analysis of apoptosis. The expression of caspase 3 differed between groups—rats from the E and RC groups exhibited higher expression, focused mostly in the subcapsular part of zona glomerulosa and along sinusoids of zona fasciculata (Figure 3). This suggests a slight, but noticeable rate of apoptosis in both the experimental and reversibility control group. P53 expression was similarly low in all groups, which suggests p53-independent apoptosis (Figure 4).

**Figure 5:**
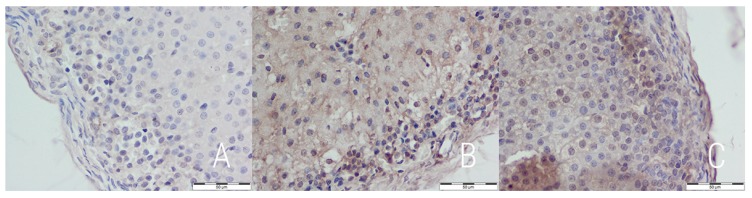
CTGF expression in adrenal glands 400× magnified in the control (**A**), experimental (**B**) and reversibility control (**C**) groups. Visible insular and irregular expression. IHC staining (bar = 50 µm).

A Ki-67 immunohistochemical staining was performed to assess the mitotic activity. No significant differences in the percentage of Ki-67 positive nuclei were noted between the groups (Table 5). For further analysis of fibrosis, an immunohistochemical staining against CTGF was prepared. A significantly higher expression of CTGF was observed in both the experimental and reversibility control groups. The pigment was distributed unevenly, forming insular structures (Figure 5). The overall picture of CTGF expression matched the Masson’s trichrome.

## 4. Discussion

In the current study, the experimental group was constantly under the influence of energy drink ingredients, mostly caffeine and taurine. Caffeine has been reported to increase serum concentration of cortisol [16,21,22], which is most probably connected with the activation of the hypothalamus-pituitary-adrenal glands axis with consequent release of ACTH and cortisol [21]. Marzouk et al. have reported that caffeine accelerates the rate of hypothalamus-pituitary-adrenal gland axis recovery in rats after chronic exposure to exogenous corticosteroids [23]. *Zona fasciculata* is the main source of corticosterone while *zona glomerulosa* is the 2nd producer of corticosterone [24]. Therefore, the decrease in lipid droplets contribution to the total area and the increase in the contribution of *zona fasciculata* to total cortex thickness is probably a sign of high glucocorticosteroid production. What is important, as another study has shown, a short period of high stress level causes severe decrease in the area occupied by lipid droplets and the weight of *zona fasciculata* in rats’ adrenal glands [25].

What is important, the once up-regulated glucocorticosteroid secretion and related cellular and histological changes in the adrenal cortex by the chronic exposure to energy drinks are potentially reversible. The results of the current study even suggest that the cessation of the activating influence of caffeine leads to at least partial reversion to the state prior to exposure. It is most noticeable in *zona fasciculata*, which is understandable, as the endocrine function and even survival of cells in this layer are most strictly related to ACTH level. It can be explained on the basis of a decrease in ACTH level caused by the cessation of stimulating action of caffeine on the hypothalamic-pituitary-adrenal [HPA] axis [26]. The ACTH is an essential factor for survival and biological activity of adrenal cortex cells, especially those of *zona fasciculata* and *zona reticularis*. Sudden cessation of the energy drink consumption is also the most probable reason for the observed increased vacuolization in *zona fasciculata* of the RC group. This may be related to cell involution because of insufficient trophic stimulation.

The increased excretory function of adrenal glands in rats chronically exposed to energy drinks may be partially related also to taurine—according to Sapronov et al., taurine derivatives increase cortisol secretion. [27]. It has lately been reported that taurine itself causes increase in corticosterone levels in rats [28]. Taurine also affects the renin-angiotensine-aldosterone axis by promoting the expression of ACE2 over ACE and thus lowering the angiotensine activity [29]. Garcia et al. have found that taurine-containing energy drink consumption may cause an increase in human salivary cortisol level [30]. The effects of energy drink consumption on the salivary cortisol levels may depend on the exact composition of the drink, including some additives. No difference has been noted in salivary cortisol levels in firefighters who performed physical training after taurine-free energy drink consumption [31]. Therefore, taurine seems an important factor in the stimulation effect which energy drinks exert on glucocorticoid production.

Our conclusions are further supported by the observed increase in the contribution of *zona fasciculata* to total cortex thickness in both the experimental and reversibility control groups [the highest values in the experimental group], which is consistent with the increased excretory function of adrenal glands and related hypertrophy and hyperplasia. Not only the cortex seems to be overstimulated by energy drinks; signs of weariness have also been observed. The number of pyknotic nuclei and the intensity of caspase 3 staining indicate a slightly elevated rate of apoptosis in both the experimental and reversibility control groups. Along with accumulation of collagen, this suggests significant overstimulation of cortical cells, which in turn leads to their damage and apoptotic death after energy drink cessation.

The current study has an important limitation. It is a purely histological evaluation and is not accompanied by any direct functional assessment (like corticosterone measurement) or adrenal gland weight measurement.

## 5. Conclusions

Chronic consumption of energy drinks seems to significantly stimulate adrenal cortex, mostly *zona fasciculata*. The results of the current study suggest that the stimulation is so intense that it cause significant damage to cortical cells, resulting in their apoptosis. It seems, however, that the observed effects are at least partially reversible. The current study lacks, however, any functional assessment. Future studies should include direct steroid measurement.

To the authors’ knowledge it is the first study on histopathological changes within adrenal glands related to chronic energy drinks consumption.

## Figures and Tables

**Table 1:** The contribution of lipid droplets to the total selected area within different layers of cortex.

Cortex Layer	Control Group	Experimental	Reversibility Control	*p* Value ^A^
Zona glomerulosa, %	14.89 ± 9.28	11.69 ± 5.02	8.32 ± 3.37	0.1097
Zona fasciculata, %	21.10 ± 4.12	11.77 ± 3.62	15.25 ± 5.20	0.0008
Zona reticularis, %	3.78 ± 1.91	8.9 ± 5.22	6.85 ± 2.88	0.0061

The values are expressed as mean ± SD. ^A^—*F* variance analysis test.

**Table 2:** The mean percentage of pyknotic nuclei.

Cortex Layer	Control Group	Experimental	Reversibility Control	*p* Value ^A^
Zona glomerulosa, %	1.38 (1.18 to 1.57)	1.96 (1.90 to 2.03)	0.80 (0.73 to 1.14)	0.07
Zona fasciculata, %	1.30 (1.11 to 1.49)	1.17 (0.49 to 2.92)	0.54 (0.54 to 1.43)	0.75
Zona reticularis, %	0.53 (0.43 to 0.63)	2.52 (2.42 to 2.62)	1.09 (0.67 to 1.72)	0.07

The values are expressed as median (interquartile range). ^A^—Kruskal-Wallis test.

**Table 3:** The contribution of cortex layers to cortex total thickness.

Cortex Layer	Control Group	Experimental	Reversibility Control	*p* Value ^A^
Zona glomerulosa, %	8.07 (7.49 to 8.83)	4.72 (3.96 to 5.30)	6.89 (5.70 to 7.66)	<0.000001
Zona fasciculata, %	50.19 (46.97 to 53.70)	55.81 (50.03 to 58.87)	50.40 (46.63 to 54.01)	0.015
Zona reticularis, %	41.90 (38.92 to 44.40)	40.43 (36.10 to 44.63)	42.01 (38.92 to 47.34)	0.586

The values are expressed as median (interquartile range). ^A^—Kruskal-Wallis test.

**Table 4:** The mean optical density (a.u.) of slides stained according to Masson’s trichrome. Higher values account for more intense fibrosis.

Cortex Layer	Control Group	Experimental	Reversibility Control	*p* Value ^A^
Zona glomerulosa	0.087 (0.083 to 0.090)	0.192 (0.174 to 0.496)	0.152 (0.132 to 0.293)	<0.00001
Zona fasciculata	0.110 (0.884 to 0.119)	0.260 (0.186 to 0.645)	0.156 (0.145 to 0.322)	0.0001
Zona reticularis	0.112 (0.102 to 0.130)	0.200 (0.176 to 0.213)	0.186 (0.176 to 0.384)	0.009

The values are expressed as median (interquartile range). ^A^—Kruskal-Wallis test.

**Table 5:** The mean percentage of Ki-67 positive nuclei.

Cortex Layer	Control group	Experimental	Reversibility Control	*p* Value ^A^
Zona glomerulosa, %	5.21 (4.12 to 6.30)	6.68 (4.74 to 8.63)	3.20 (1.63 to 4.55)	0.26
Zona fasciculata, %	5.71 (4.46 to 6.97)	8.56 (2.93 to 8.75)	4.29 (4.27 to 4.58)	0.47
Zona reticularis, %	8.44 (6.62 to 10.26)	3.93 (3.34 to 4.46)	4.32 (4.04 to 4.60)	0.16

The values are expressed as median (interquartile range). ^A^—Kruskal-Wallis test.
